# Comparative genetic and epigenetic diversity in pairs of sympatric, closely related plants with contrasting distribution ranges in south-eastern Iberian mountains

**DOI:** 10.1093/aobpla/plaa013

**Published:** 2020-04-08

**Authors:** Mónica Medrano, Conchita Alonso, Pilar Bazaga, Esmeralda López, Carlos M Herrera

**Affiliations:** Estación Biológica de Doñana, Consejo Superior de Investigaciones Científicas (CSIC), Isla de La Cartuja, Sevilla, Spain

**Keywords:** AFLP, DNA methylation, endemism, epigenetic diversity, genetic diversity, Mediterranean mountains, MSAP, population epigenetics

## Abstract

Genetic diversity defines the evolutionary potential of a species, yet mounting evidence suggests that epigenetic diversity could also contribute to adaptation. Elucidating the complex interplay between genetic and epigenetic variation in wild populations remains a challenge for evolutionary biologists, and the intriguing possibility that epigenetic diversity could compensate for the loss of genetic diversity is one aspect that remains basically unexplored in wild plants. This hypothesis is addressed in this paper by comparing the extent and patterns of genetic and epigenetic diversity of phylogenetically closely related but ecologically disparate species. Seven pairs of congeneric species from Cazorla mountains in south-eastern Spain were studied, each pair consisting of one endemic, restricted-range species associated to stressful environments, and one widespread species occupying more favourable habitats. The prediction was tested that endemic species should have lower genetic diversity due to population fragmentation, and higher epigenetic diversity induced by environmental stress, than their widespread congeners. Genetic (DNA sequence variants) and epigenetic (DNA cytosine methylation variants) diversities and their possible co-variation were assessed in three populations of each focal species using amplified fragment length polymorphism (AFLP) and methylation-sensitive AFLP (MSAP). All species and populations exhibited moderate to high levels of genetic polymorphism irrespective of their ecological characteristics. Epigenetic diversity was greater than genetic diversity in all cases. Only in endemic species were the two variables positively related, but the difference between epigenetic and genetic diversity was greater at populations with low genetic polymorphism. Results revealed that the relationship between genetic and epigenetic diversity can be more complex than envisaged by the simple hypothesis addressed in this study, and highlight the need of additional research on the actual role of epigenetic variation as a source of phenotypic diversity before a realistic understanding of the evolutionary relevance of epigenetic phenomena in plant adaptation can be achieved.

## Teaser

Genetic diversity is generally considered the chief determinant of evolutionary change, but epigenetic diversity is now recognized as another layer of heritable variation with potential adaptive consequences. Epigenetic diversity could sometimes (fragmented populations, stressing habitats) alleviate the loss of genetic diversity and provide an ‘evolutionary backup’ mechanism for wild plants. This study compares genetic and epigenetic diversity in seven congeneric species pairs with restricted and widespread distributions in south-eastern Spain. Results suggest that higher epigenetic diversity could alleviate the loss of genetic diversity in some populations of endemic plants, but also other plant features can be essential to understand the relationship between genetic and epigenetic diversities.

## Introduction

The genetic diversity of species and populations has multiple implications for their ecology, evolution and survival. For example, reduced genetic diversity arising from inbreeding, fragmentation, bottlenecks or founder effects have been long known to pose threats on long-term survival of species and populations (Ellstrand and Elam 1993; Frankham 2005; Allendorf and Luikart 2007; Allendorf 2017). An increasing number of recent studies are showing, however, that genetic diversity (i.e. depending on variation in DNA nucleotide sequence) is not the only heritable genomic information that could influence the ecology, evolution or survival of populations. Epigenetic variations that depend on DNA methylation or chromatin states can influence phenotypic traits and are often inherited over generations in plant populations (Richards 2006; Verhoeven *et al.* 2010; Quadrana and Colot 2016; Herrera *et al.* 2018). One defining feature that sets epigenetic variation apart from genetic variation is the capacity to exhibit modifications in response to environmental factors (see, e.g., Dowen *et al.* 2012; Alonso *et al.* 2016).

There is now a growing consensus that natural epigenetic diversity could endow wild plant populations with an extra layer of heritable phenotypic variation that could complement genetically based variation and contribute to local adaptation and survival (Grativol *et al.* 2012; Medrano *et al.* 2014; Schulz *et al.* 2014; Herrera *et al.* 2017; Gáspár *et al.* 2019; but see Herden *et al.* 2019). Compelling evidence supporting a role for epigenetic variation as an additional component of genomic variation has been provided by studies showing, for instance, that in wild plant populations epigenetic diversity is often greater than genetic variation (Herrera *et al.* 2016, 2017; and references therein) and can compensate for the complete or nearly complete loss of genetic variation in small colonizing populations, apomictic species or genetically homogeneous clones (Richards *et al.* 2012; Spens and Douhovnikoff 2016; Wilschut *et al.* 2016; Guarino *et al.* 2019; Jueterbock *et al.* 2019; Shi *et al.* 2019; Wang *et al.* 2020). Interestingly, in experimental populations of *Arabidopsis thaliana* it has been recently shown that epigenetic variation is under selection and contributes to rapid phenotypic adaptive responses in absence of consistent genetic changes (Schmid *et al.* 2018). More studies with non-model plants growing in a real-world context are essential to understand the potential role of epigenetic variation in plant adaptation and in ecological and evolutionary processes.

Narrowly endemic plants provide a hitherto unexplored study system particularly well suited for assessing the hypothesis that epigenetic diversity could mitigate the loss of genetic diversity in wild plants. On one side, narrow endemics with restricted distributions often have small, discrete, isolated populations with lower levels of genetic diversity than close relatives with broader geographical distributions (Kruckeberg and Rabinowitz 1985; Cole 2003). And on the other hand, at least in the Mediterranean, endemic plants often live in stressful environments, ecologically marginal habitats and/or highly specific habitat disturbance regimes (Lavergne *et al.* 2004; Thompson *et al.* 2005; Totté *et al.* 2015). Since biotic and abiotic stresses (e.g. herbivory, aridity, extreme temperatures) are able to induce heritable epigenetic modifications in plant genomes (Alonso *et al.* 2016; Quadrana and Colot 2016), the association of endemic plants with stressful environments could in itself promote epigenetic diversity via environmental induction of epigenetic variants. Recent models suggest that, when populations are small, epigenetic variation would particularly promote adaptation by rapidly restoring rare adaptive states that would otherwise be lost by genetic drift, or in divergent, peripheral environments, especially when epimutations are adaptively biased (Smithson *et al.* 2019).

This study examines comparative patterns of genetic and epigenetic diversity in wild populations of seven congeneric species pairs occurring sympatrically in the Sierra de Cazorla mountain range, one important glacial refuge and plant biodiversity hotspot in south-eastern Spain (Médail and Quézel 1999; Médail and Diadema 2009), associated to various habitat types. Specifically, we test the expectation that endemic plants with restricted distributions should have lower genetic diversity but higher epigenetic diversity than widespread ones. The paired-species approach used here has been often applied in previous comparisons of ecological, biological or genetic features of endemic and widespread species, as it allows to control for possible phylogenetic effects on interspecific differences (e.g. Karron 1987; Lavergne *et al.* 2004). By analysing pairs in seven different genera within the same region we also controlled for geographic effects. One novelty of our sampling design was the concurrent estimation of genetic and epigenetic diversity in replicated population samples of each species in each congeneric pair. In this way, robust statistical tests of hypothesized relationships between genetic and epigenetic diversity could be undertaken by applying linear mixed models to the data and treating plant species and populations as random effects. Linear mixed models allow drawing conclusions with reference to a broad inference space, which makes them particularly well suited to answer questions whose scope transcends the limits of the particular samples studied (Bolker *et al.* 2009; Bolker 2015). Specifically, we expected that (i) epigenetic diversity should be greater than genetic diversity at population level; (ii) after controlling by species relatedness and geographic location, endemic species with restricted distribution should have lower genetic diversity and higher epigenetic diversity than their widespread congeners. If so, then (iii) genetic and epigenetic diversities should have an inverse relationship particularly significant across populations of endemic plants.

## Methods

### Site and study species

**Figure 1.**
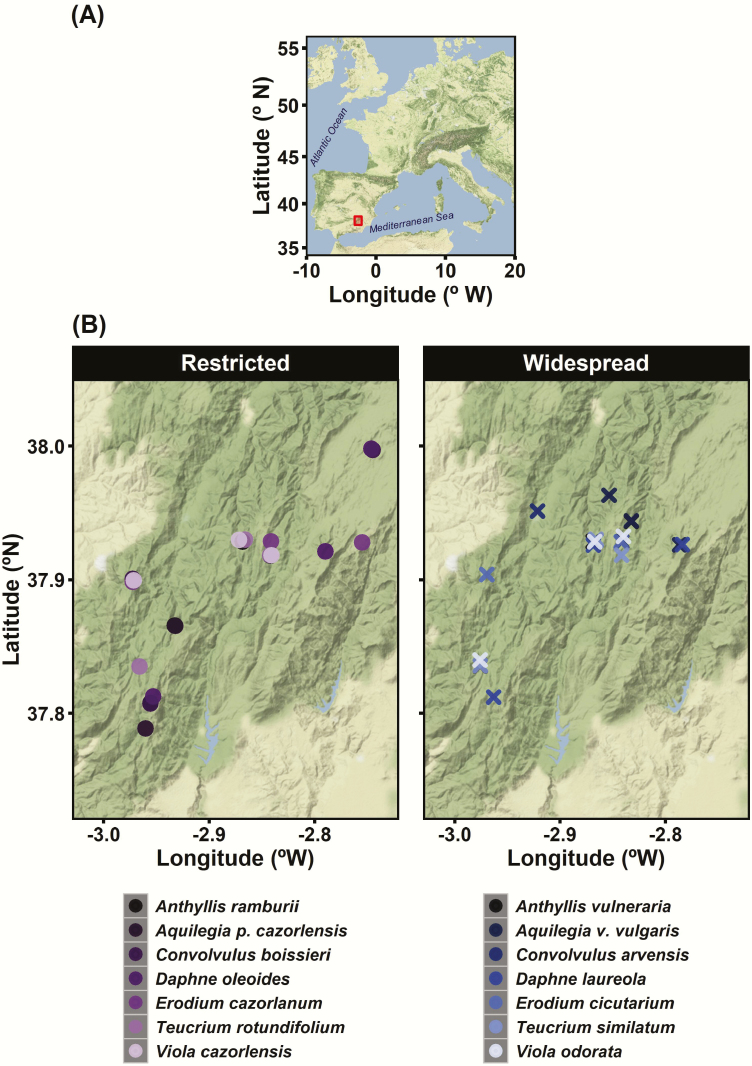
Geographical location of our study site, the Natural Park of Sierras de Cazorla Segura and Las Villas (red rectangle), in south-eastern Iberian Peninsula (A). Maps showing the approximate location of the 21 populations from the seven restricted endemic species (left panel), and the 21 populations from the seven widespread congeners (right panel) included in this study, showing that they were similarly distributed across the study area (B).

Field sampling for this study was carried out in Sierras de Cazorla-Segura-Las Villas Natural Park (Jaén Province, Spain), one of several mountain units comprising the Baetic Ranges complex in south-eastern Iberian Peninsula (Fig. 1A). This Natural Park is the largest protected space in Spain with a total area of 209 920 ha and altitudes ranging from 590 to 2107 m above sea level. The region is characterized by rugged topography, large expanses of well-preserved habitats, a large proportion of endemic species and outstanding floristic diversity (Médail and Quézel 1999; Mota *et al.* 2002; Melendo *et al.* 2003).

**Figure 2.**
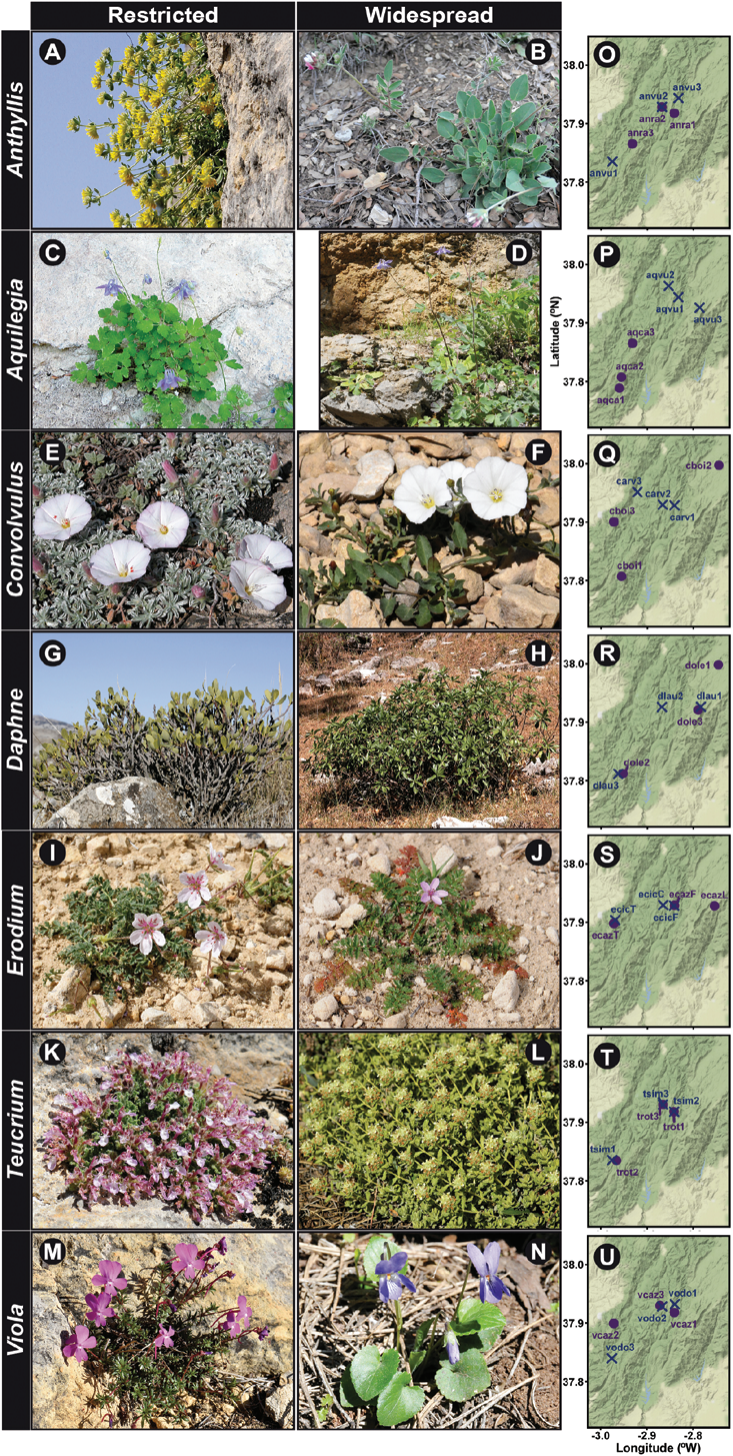
Photographs showing the general aspect of flowering individuals from each of the 14 study species (A–N), grouped by genera: the restricted species of each genus always on the left and the widespread on the right. Maps showing the exact location of the three populations sampled from each pair of study species (O–U). *Anthyllis ramburii* (A), *Anthyllis vulneraria* (B), *Aquilegia p. cazorlensis* (C), *Aquilegia v. vulgaris* (D), *Convolvulus boissieri* (E), *Convolvulus arvensis* (F), *Daphne oleoides* (G), *Daphne laureola* (H), *Erodium cazorlanum* (I), *Erodium cicutarium* (J), *Teucrium rotundifolium* (K), *Teucrium similatum* (L), *Viola cazorlensis* (M), *Viola odorata* (N). Population names refer to Table 1. In each map purple points represent populations of restricted species while blue crosses populations of its widespread congener.

For the present study we selected seven congeneric species pairs. Each pair consisted of one narrow endemic species with a restricted geographic distribution and that is a specialist of stressing Mediterranean microhabitats or that occurs only in highly specific habitats, and one species with a widespread geographic distribution which utilizes a broad range of habitat types (see Table 1 and Fig. 2 for more details). The seven pairs of restricted and widespread selected species, mentioned in this order, were: *Anthyllis ramburii* and *Anthyllis vulneraria* (Fabaceae, Fig. 2A and B); *Aquilegia pyrenaica* subsp*. cazorlensis* and *Aquilegia vulgaris* subsp. *vulgaris* (Ranunculaceae, Fig. 2C and D); *Convolvulus boissieri* and *Convolvulus arvensis* (Convolvulaceae, Fig. 2E and F); *Daphne oleoides* and *Daphne laureola* (Thymelaeaceae, Fig. 2G and H); *Erodium cazorlanum* and *Erodium cicutarium* (Geraniaceae, Fig. 2I and J); *Teucrium rotundifolium* and *Teucrium similatum* (Lamiaceae, Fig. 2K and L); and *Viola cazorlensis* and *Viola odorata* (Violaceae, Fig. 2M and N). All except *E. cicutarium* are perennial plants. In addition, within the genera *Anthyllis*, *Convolvulus*, *Erodium* and *Viola* the restricted species has woody stocks, whereas the widespread congener is herbaceous. Data on species distribution and habitat requirements were obtained from Flora Iberica (Castroviejo *et al.* 1986–2012), Flora Vascular de Andalucía Oriental (Blanca *et al.* 2011) and Proyecto Anthos (http://www.anthos.es/; Aedo and Castroviejo 2005).

**Table 1.** Description of the habitat type and geographic distribution for the 14 species included in this study, and details of the location (longitude and latitude), altitude and sample sizes (*N*) for each of the three populations sampled per species. Longitudinal and latitudinal coordinates are given as decimal coordinates (WGS84). ^R^restricted distribution; ^W^widespread distribution.

Taxa	Habitat type	Distribution	Population	Longitude	Latitude	Altitude (m asl)	*N*
*Anthyllis ramburii* ^R^	High mountain dwarf procumbent scrubs, sandy dolomitic soils	Baetic Ranges	anra1	−2.84164	37.91791	1460	25
			anra2	−2.86832	37.92836	1636	25
			anra3	−2.93244	37.86575	1473	25
*Anthyllis vulneraria* ^W^	Dry grasslands and rocky environments with calcareous soils, broad altitudinal range	European, Mediterranean basin east to the Caucasus	anvu1	−2.97559	37.83554	1402	25
			anvu2	−2.86850	37.92843	1645	25
			anvu3	−2.83235	37.94364	1332	25
*Aquilegia p. cazorlensis* ^R^	Rifts of limestone outcrops, sandy soils in shady, damp sites at cliff bases	Baetic Ranges	aqca1	−2.96022	37.78849	1495	25
			aqca2	−2.95573	37.80753	1901	25
			aqca3	−2.93202	37.86538	1475	25
*Aquilegia v. vulgaris* ^W^	Stream margins, poorly drained open meadows around springs, broad altitudinal range	European temperate element and Mediterranean	aqvu1	−2.83231	37.94395	1356	25
			aqvu2	−2.85351	37.96322	1186	25
			aqvu3	−2.78665	37.92610	1713	25
*Convolvulus boissieri* ^R^	High mountain dwarf procumbent scrubs, sandy dolomitic soils	Baetic Ranges	cboi1	−2.95572	37.80722	1919	25
			cboi2	−2.74480	37.99704	1711	28
			cboi3	−2.97231	37.90047	1535	25
*Convolvulus arvensis* ^W^	Cultivated areas, wasteland, roadsides, grassy slopes, broad altitudinal range	Temperate and tropical regions, except Australia	carv1	−2.84134	37.92845	1511	25
			carv2	−2.86664	37.92967	1630	25
			carv3	−2.92134	37.95132	818	25
*Daphne oleoides* ^R^	Calcareous xerophytic woodlands and scrublands, rocky slopes	North Africa, southern Europe, Asia Minor	dole1	−2.74612	37.99791	1725	25
			dole2	−2.95303	37.81240	1931	25
			dole3	−2.78965	37.92117	1703	25
*Daphne laureola* ^W^	Sclerophyllous and semi-deciduous forests, understory of montane forests, mostly in basic soils	Palaearctic	dlau1	−2.78407	37.92632	1653	25
			dlau2	−2.86837	37.92602	1629	25
			dlau3	−2.96303	37.81203	1774	25
*Erodium cazorlanum* ^R^	High mountain dry grasslands and rocky environments, sandy dolomitic soils	Baetic Ranges	ecazF	−2.84126	37.92870	1526	32
			ecazL	−2.75469	37.92780	1754	33
			ecazT	−2.97236	37.89824	1598	40
*Erodium cicutarium* ^W^	Meadows, flood plains, gravel areas, roadsides and disturbed areas, broad altitudinal range	Eurosiberian Southern-temperate element, widely naturalized now Circumpolar	ecicC	−2.86574	37.92942	1622	28
			ecicF	−2.96944	37.90388	1541	30
			ecicT	−2.84219	37.92835	1520	23
*Teucrium rotundifolium* ^R^	Rocky outcrops	Iberian Peninsula–Morocco	trot1	−2.84209	37.91830	1466	25
			trot2	−2.96602	37.83489	1475	25
			trot3	−2.86583	37.93001	1637	25
*Teucrium similatum* ^W^	Sclerophyllous and semi-deciduous forests, calcareous scrublands and grasslands, rocky mountain slopes, broad altitudinal range	Iberian Peninsula	tsim1	−2.97571	37.83560	1410	25
			tsim2	−2.84163	37.91844	1441	25
			tsim3	−2.86590	37.93026	1622	25
*Viola cazorlensis* ^R^	Rocky outcrops, cliffs, sandy dolomitic soils	Baetic Ranges	vcaz1	−2.84075	37.91833	1437	25
			vcaz2	−2.97145	37.89952	1537	25
			vcaz3	−2.87158	37.92985	1600	25
*Viola odorata* ^W^	Open woodlands, hedge banks and scrublands, edges of forests and clearings, broad altitudinal range	Europe and Asia	vodo1	−2.83988	37.93238	1483	24
			vodo2	−2.86705	37.92842	1631	25
			vodo3	−2.97588	37.83988	1350	25

### Field sampling

In order to assess intra- and interspecific patterns of genetic and epigenetic diversity we sampled three populations per species and in each population 23–40 widely spaced flowering individuals (Table 1, Fig. 1B, Fig. 2O and U). A total of 1088 individuals were sampled for the 14 species included in this study. The sampling design aimed to fulfil the appropriate number of individuals analysed to obtain right estimates of population diversities (Nybom 2004) and a balanced sample for the comparisons across species within a specific geographic location. Young leaves were collected from each plant, placed in paper envelopes and dried at ambient temperature in containers with silica gel. Field collections were carried out during the flowering season (April to June) of each studied taxa.

### Laboratory methods

Total genomic DNA was extracted from dry leaf material of all plants sampled using ISOLATE II Plant DNA Kit (Bioline, London, UK) and the manufacturer’s protocol. Genetic and epigenetic analyses were conducted on the same DNA extracts.

Genetic fingerprints were obtained for each plant using amplified fragment length polymorphism (AFLP) markers (Vos *et al.* 1995; Weising *et al.* 2005; Meudt and Clarke 2007). The AFLP analyses were performed using standard protocols involving the use of fluorescent dye-labelled selective primers (Weising *et al.* 2005). After testing different combinations of selective primer pairs, four different combinations of *Mse*I + 3/*Pst*I + 2 primer pairs that resolved more reproducible and easier to score bands were selected on each genus and AFLP analyses were conducted using them (**see****Supporting Information—Table S1** for a complete overview of all primers used for each genus with the AFLP protocol. Note that the comparisons between species within genera were done using the same four primer pairs).

Plants were also characterized epigenetically using the methylation-sensitive amplified polymorphism (MSAP) technique (Reyna-López *et al.* 1997; Schulz *et al.* 2013; Fulneček and Kovařik 2014; Guevara *et al.* 2017). Methylation-sensitive amplified polymorphism is a modification of the standard AFLP technique that uses the methylation-sensitive restriction enzymes *Hpa*II and *Msp*I in parallel runs in combination with another restriction enzyme (here the frequent cutter *Mse*I is used). *Hpa*II and *Msp*I are two isoschizomers that recognize and cleave the same tetranucleotide sequence 5′-CCGG, but differ in their sensitivity to the methylation state of cytosine. *Hpa*II can cut at sites that are either non-methylated or contain one methylated external cytosine, whereas *Msp*I cuts non-methylated sites and those with one or two methylated internal cytosine (see Schulz *et al.* 2013; Fulneček and Kovařic 2014 for further details). Namely, both enzymes cut the DNA if the restriction site is not methylated, but they cut in a different way in the presence of cytosine methylation. After testing different combinations of selective primer pairs three or four *Mse*I + 3/*Hpa*II–*Msp*I + 2 primer combinations that resolved more reproducible and easier to score bands were selected on each genus and MSAP assays were conducted using them (**see****Supporting Information—Table S1** for a complete overview of the primers used in each genus for the MSAP protocol. Again, the same primer pairs were used for the two species within each genus).

Amplified products from both AFLP and MSAP protocols were analysed on an ABI PRISM 3130xl DNA sequencer, and fingerprint profiles were scored manually by visualizing electrophoregrams with GeneMapper 5.0 software. All primer combinations used with each species were merged in a single binary data table to generate finally one genetic (AFLP) and two epigenetic (MSAP) raw data matrices (see below). We assessed the repeatability of banding patterns for each species by repeating the entire AFLP/MSAP protocol in a number of randomly selected samples. For each species an average of 11.2 % (AFLP) and 25.8 % (MSAP) of the samples were used as replicates (for further details, **see****Supporting Information—Table S2**). After elimination of inconsistent bands, overall error rates ranged from 1.05 to 3.48 % and from 2.79 to 4.85 %, respectively, for AFLP and MSAP raw data sets **[see****Supporting Information—Table S2****]**. In the whole group of 1088 plants a total of 2918 AFLP markers were scored, with a mean of 208.4 markers scored per plant (range = 86–418).

Some of the greatest advantages of AFLP and MSAP techniques are their wide genome sampling, high reproducibility and ability to generate many polymorphic bands per reaction without prior knowledge of genomic sequence of the organisms being assayed (Meudt and Clarke 2007; Paun and Schönswetter 2012). On the contrary, one of the most important disadvantages of these two techniques is that bands obtained are considered dominant, implying that polymorphism is scored only in terms of presence or absence, and thus it is not viable in any of them to distinguish between individuals being heterozygous or homozygous for the dominant allele (Paun and Schönswetter 2012). Although different attempts of codominant scoring using band intensities have been proposed (see, for instance, Gort and van Eeuwijk 2010; Foll *et al.* 2010), various drawbacks, like high unreliable genotype assignments or important loss of information, advised against the regular use of those approaches to obtain codominant markers. For typical population diversity studies this problem can be at least partially mitigated by the high number of polymorphisms that are generated per reaction (Paun and Schönswetter 2012) and by sampling a large number of individuals per population (Lynch and Milligan 1994), as we have done here. Another important limitation of these two techniques is that the sequence content of each AFLP or MSAP marker remains unknown throughout the whole process, i.e. they are anonymous markers, restricting their usefulness to the description of population patterns but obstructing it for further analyses. Although new developments in next-generation sequencing technologies are offering affordable ways to overcome marker anonymity, adopting this approach in a study like the present, with such a high number of individuals and populations from quite a few non-model species, is nowadays still unfeasible in terms of money and time. A specific drawback of the MSAP technique is that it only detects differences in methylation that occur at the restriction sites of the cutter endonucleases, underestimating the overall level of methylation (Schrey *et al.* 2013; Fulneček and Kovařik 2014; Alonso *et al.* 2016). In spite of this limitation, MSAP markers have been validated as an alternative to whole-genome bisulfite sequencing (WGBS) (Lauria *et al.* 2017) and proven useful to investigate variation in genome-wide patterns of cytosine methylation in non-model plants that lack sequenced genomes (Medrano *et al.* 2014; Foust *et al.* 2016; Herrera *et al.* 2016; Wilschut *et al.* 2016; Thiebaut *et al.* 2019). In particular, for comparative interspecific studies of non-model organisms with large sample sizes like this, the application of these two cost-effective techniques are probably the only affordable methods that currently can provide reliable, robust and relatively simple genome-wide information simultaneously on both DNA polymorphisms as well as putative changes in DNA cytosine methylation.

### Data analysis

#### DNA methylation analyses

The MSAP profiles were analysed with the R script ‘MSAP_calc’ (Schulz *et al.* 2013) using the ‘*Extract_MSAP_epigenotypes*’ function applied independently to each species with the following parameters: Epicode = ‘Mix1’, delete.monomorphic.loci = TRUE and MinPoly = 2. This software analyses the MSAP binary matrix based on four types of methylation pattern according to the presence or absence of one or both fragments of *Mse*I/*Hpa*II and *Mse*I/*Msp*I: (1) fragments present in both profiles (1/1), indicating an unmethylated state; (2) fragments present only in *Mse*I/*Hpa*II profiles (1/0), indicating hemi-methylated CHG sites; (3) fragments present only for *Mse*I/*Msp*I (0/1), indicating hemi- or fully methylated CG sites; and (4) absence of fragments in both profiles (0/0), representing an uninformative state caused either by different types of methylation, or due to restriction site polymorphism. Under the ‘Mix1’ scoring scheme the MSAP profiles are transformed into two binary data sets: one data set of methylated epiloci (hereafter M-MSAP) which scores conditions (2) and (3) as 1 and all other conditions as 0; and a data set of unmethylated epiloci (U-MSAP) which scores condition (1) as 1 and conditions (2) and (3) as 0. All plants sampled were characterized epigenetically by presence–absence scores for U- and M-type MSAP markers. In total 1450 U-MSAP and 2197 M-MSAP markers were scored in the whole group of 1088 plants sampled, with a mean per individual plant of 103.6 (range = 54–181) and 156.9 (range = 95–213), respectively, for U-MSAP and M-MSAP markers.

### Diversity indices

Binary AFLP and MSAP raw data sets were analysed following the same framework using a band-based strategy which did not require calculating allele frequencies (Bonin *et al.* 2007). Genetic (AFLP) and epigenetic (MSAP) diversity within populations were quantified using four different indices: (i) proportion of polymorphic fragments (PPOL); (ii) Shannon’s diversity index (SI); (iii) proportion of private fragments (PPRIV), i.e. bands unique of each single population; and (iv) the frequency-down-weighted marker value, in the following termed ‘rarity index’ (RI). Shannon’s diversity index (SI) was calculated for each locus within each population using the formula:

$$\begin{array}{l} SI=-\overset{}{\sum }P_{i}\cdot \log_{e}(P_{i}) \end{array}$$ (1)

where *P*_*i*_ is the frequency of the presence or absence of the band. The mean SI per population is given by an average of the index values over individual loci (Pérez-Figueroa 2013). The rarity index for individual *x* (RI_*x*_) was calculated according to Schönswetter and Tribsch (2005) using the formula:

$$\begin{array}{l} RI_{x}=\underset{i=1}{\overset{n}{\sum }}\frac{s_{ix}}{\overset{k}{\underset{j=1}{\sum }}s_{ij}} \end{array}$$ (2)

where *n* is the number of markers, *s*_*ix*_ is the binary state of the *i*th marker in individual *x* (either 1 or 0) and *k* is the total number of individuals in the population data set (i.e. within the population sample). In the denominator the number of occurrences of the *i*th marker in the total population data set is calculated. Population rarity index (RI) was estimated as the average of individual values. Calculations were carried out using ‘AFLPdat’ (function ‘*Rarity*—*rarity 1*’, Version 20.10.2010; Ehrich 2006). Altogether we finally obtained 42 population data for each diversity index and marker type.

### Statistical analysis

All statistical analyses were carried out using the R environment (R Development Core Team 2019). Differences in genetic and epigenetic diversity between restricted and widespread species were analysed using linear and generalized mixed effect models with each of the four population diversity indices (PPOL, PPRIV, SI and RI) as response variables, ‘Markers’ (with three levels: AFLP, U-MSAP and M-MSAP) and ‘Distribution’ (with two levels: restricted and widespread) and their interaction as fixed effects, and ‘Populations’ nested within ‘species’ as random effect to specify paired comparisons at population level. The ‘*lmer*’ and ‘*glmer*’ functions from the ‘lme4’ package were used to fit linear mixed and generalized linear mixed models, respectively (Bates *et al.* 2015). The number of loci was included as a weighing factor to account for variance across species and type of markers **[see****Supporting Information—Table S3****]**. In generalized linear models, proportions (PPOL, PPRIV) were modelled as binomial processes. Residuals were graphically inspected for linearity and homoscedasticity, and normal distribution of residuals was not rejected (*P* > 0.05) in any of the fitted models presented. Statistical significance of the two fixed effects and their interaction on response variables was determined in all cases with ordinary likelihood ratio tests using the ‘*anova*’ function from the R ‘stats’ library (Zuur *et al.* 2009). In each analysis, estimated marginal means (*sensu*Searle *et al.* 1980) and associated confidence intervals for the response variable at each factor level were obtained with the ‘*emmeans*’ function of the ‘emmeans’ library (Lenth 2018). *Post hoc* analysis was done by conducting multiple pairwise comparisons of the estimated marginal means with Tukey adjustment. Marginal means from generalized linear models involving proportions were back-transformed to the original scale of measurement.

The relationship between epigenetic and genetic diversity of study populations was separately explored for restricted and widespread species using correlation analyses.

## Results

### Variation in genetic and epigenetic diversity in populations of restricted and widespread plants

**Figure 3.**
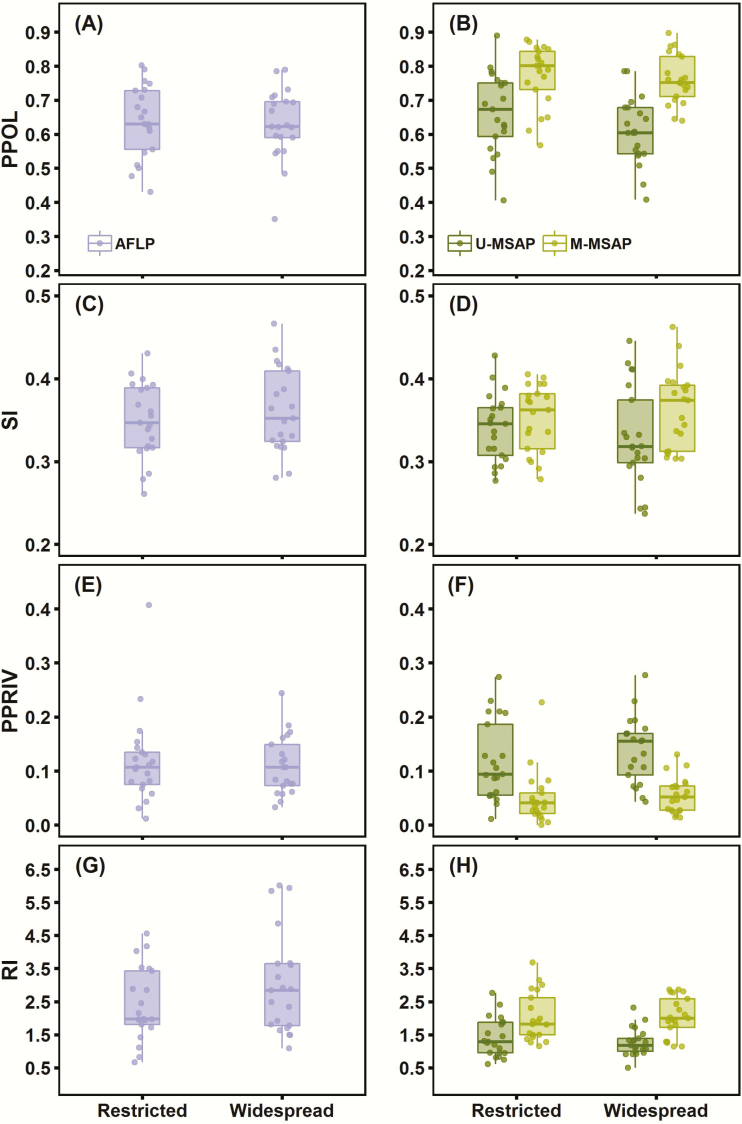
Variation in genetic (AFLP) and epigenetic (U-MSAP and M-MSAP) diversity estimates between populations of restricted and widespread species included in this study. Values of the four diversity indices: proportion of polymorphic fragments (PPOL, A–B), Shannon’s diversity index (SI, C–D), proportion of private fragments (PPRIV, E–F) and rarity index (RI, G–H) are depicted. In each figure each dot denotes a population, the lower and upper boundaries of the boxplot indicate the 25th and 75th percentiles, the horizontal line within the box marks the median and the whiskers indicate data range.

Values of genetic (AFLP) and epigenetic (U-MSAP and M-MSAP) diversity indices obtained in each population for the group of restricted and widespread species included in our study are depicted in Fig. 3 (see also **Supporting Information—Tables S3****and****S4**). Averaged values for each species are shown in Table 2. In general, all the genetic and epigenetic diversity indices varied widely and similarly in populations of restricted and widespread species, as denoted by their broad ranges of variation (Fig. 3). For AFLP markers PPOL ranged from 0.430 to 0.802 in populations of restricted species and from 0.351 to 0.789 in populations of widespread species (Fig. 3A), for U-MSAP markers PPOL varied from 0.406 to 0.890 and from 0.408 to 0.785, and for M-MSAP markers from 0.568 to 0.878 and 0.640 to 0.897, respectively, for restricted and widespread species (Fig. 3B). PPRIV per population varied from 0.012 to 0.407 (note however that this is an outlier value) in restricted species and from 0.033 to 0.244 in widespread species for AFLP markers (Fig. 3C), and from 0.277 to 0.428 and from 0.237 to 0.446 for U-MSAP markers, and from 0 to 0.227 and from 0.014 to 0.131 for M-MSAP markers, respectively, restricted and widespread species (Fig. 3E). The SI was less variable than PPOL in our data set and, thus, less informative. It fluctuated from 0.261 to 0.430 in restricted species and from 0.280 to 0.466 in widespread ones for AFLP markers (Fig. 3C), and, respectively, from 0.277 to 0.428 and from 0.237 to 0.446 for U-MSAP markers, and from 0.278 to 0.405 and from 0.303 to 0.462 for M-MSAP markers (Fig. 3D). Rarity index ranged from 0.66 to 4.55 in populations of restricted species and from 1.08 to 6.01 in populations of widespread ones for AFLP markers (Fig. 3G), and from 0.61 to 2.76 and from 0.50 to 2.31 for U-MSAP markers, and from 1.16 to 3.68 and 1.14 to 2.86 for M-MSAP markers, respectively, in restricted and widespread species (Fig. 3H).

**Table 2.** Summary of the genetic (AFLP) and epigenetic (U-MSAP and M-MSAP) diversity estimates obtained per species. Means and standard errors (in parenthesis) are shown for proportion of polymorphic fragments (PPOL), Shannon’s diversity index (SI), proportion of private fragments (PPRIV) and rarity index (RI). ^R^restricted distribution; ^W^widespread distribution.

	PPOL			SI			PPRIV			RI		
Species	AFLP	U-MSAP	M-MSAP	AFLP	U-MSAP	M-MSAP	AFLP	U-MSAP	M-MSAP	AFLP	U-MSAP	M-MSAP
*Anthyllis ramburii* ^R^	0.707 (0.023)	0.593 (0.020)	0.769 (0.010)	0.364 (0.013)	0.340 (0.019)	0.382 (0.006)	0.104 (0.020)	0.147 (0.019)	0.069 (0.024)	1.96 (0.01)	1.15 (0.10)	1.61 (0.10)
*Anthyllis vulneraria* ^W^	0.647 (0.043)	0.604 (0.000)	0.855 (0.006)	0.295 (0.012)	0.312 (0.011)	0.389 (0.007)	0.118 (0.025)	0.168 (0.006)	0.023 (0.004)	3.12 (0.26)	1.35 (0.02)	2.72 (0.07)
*Aquilegia p. cazorlensis* ^R^	0.595 (0.068)	0.556 (0.076)	0.759 (0.056)	0.377 (0.019)	0.333 (0.012)	0.369 (0.019)	0.127 (0.053)	0.176 (0.067)	0.038 (0.018)	2.17 (0.34)	1.41 (0.31)	2.55 (0.33)
*Aquilegia v. vulgaris* ^W^	0.614 (0.035)	0.550 (0.008)	0.680 (0.019)	0.342 (0.009)	0.416 (0.016)	0.439 (0.013)	0.093 (0.041)	0.169 (0.032)	0.068 (0.023)	2.01 (0.25)	1.11 (0.09)	2.03 (0.16)
*Convolvulus boissieri* ^R^	0.656 (0.090)	0.678 (0.073)	0.762 (0.039)	0.394 (0.034)	0.382 (0.033)	0.369 (0.018)	0.099 (0.032)	0.085 (0.025)	0.064 (0.010)	2.97 (0.38)	1.61 (0.23)	2.09 (0.17)
*Convolvulus arvensis* ^W^	0.616 (0.066)	0.739 (0.027)	0.770 (0.064)	0.367 (0.012)	0.403 (0.014)	0.372 (0.011)	0.129 (0.027)	0.068 (0.010)	0.046 (0.020)	2.38 (0.31)	1.90 (0.06)	2.48 (0.35)
*Daphne oleoides* ^R^	0.0529 (0.099)	0.627 (0.059)	0.784 (0.024)	0.366 (0.029)	0.309 (0.015)	0.373 (0.015)	0.132 (0.037)	0.136 (0.034)	0.023 (0.005)	1.52 (0.22)	0.79 (0.14)	1.93 (0.08)
*Daphne laureola* ^W^	0.554 (0.101)	0.747 (0.022)	0.772 (0.035)	0.314 (0.028)	0.303 (0.009)	0.306 (0.007)	0.159 (0.125)	0.068 (0.012)	0.011 (0.006)	1.15 (0.41)	0.72 (0.06)	1.27 (0.06)
*Erodium cazorlanum* ^R^	0.0757 (0.039)	0.724 (0.026)	0.864 (0.010)	0.410 (0.011)	0.360 (0.007)	0.394 (0.007)	0.064 (0.027)	0.103 (0.009)	0.027 (0.004)	1.56 (0.31)	0.99 (0.05)	1.40 (0.07)
*Erodium cicutarium* ^W^	0.629 (0.048)	0.0639 (0.021)	0.707 (0.034)	0.429 (0.019)	0.303 (0.005)	0.309 (0.003)	0.091 (0.024)	0.111 (0.024)	0.087 (0.012)	1.60 (0.11)	1.04 (0.06)	1.23 (0.05)
*Teucrium rotundifolium* ^R^	0.675 (0.025)	0.541 (0.074)	0.762 (0.002)	0.327 (0.005)	0.241 (0.002)	0.306 (0.002)	0.111 (0.014)	0.187 (0.053)	0.056 (0.003)	5.57 (0.36)	1.73 (0.34)	2.84 (0.02)
*Teucrium similatum* ^W^	0.636 (0.006)	0.520 (0.015)	0.794 (0.034)	0.317 (0.001)	0.288 (0.006)	0.351 (0.014)	0.132 (0.014)	0.217 (0.007)	0.047 (0.017)	4.25 (0.16)	1.33 (0.06)	2.48 (0.35)
*Viola cazorlensis* ^R^	0.596 (0.021)	0.764 (0.083)	0.678 (0.089)	0.292 (0.010)	0.357 (0.016)	0.293 (0.007)	0.112 (0.006)	0.048 (0.024)	0.110 (0.059)	3.48 (0.03)	2.41 (0.20)	2.47 (0.61)
*Viola odorata* ^W^	0.657 (0.064)	0.627 (0.097)	0.776 (0.062)	0.378 (0.006)	0.341 (0.017)	0.355 (0.011)	0.126 (0.059)	0.115 (0.044)	0.065 (0.034)	4.26 (0.84)	1.25 (0.18)	1.44 (0.23)

**Figure 4.**
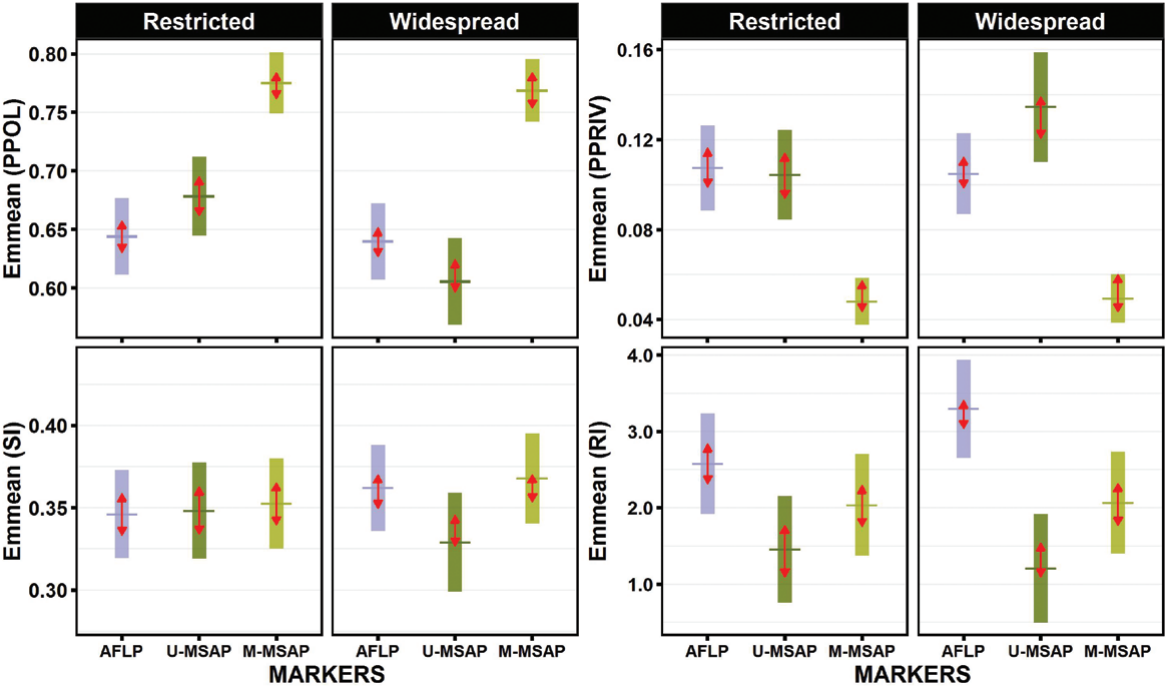
Comparisons of model-estimated marginal means (EMMs) in restricted and widespread species between genetic (AFLP) and epigenetic (U-MSAP and M-MSAP) markers obtained for the four diversity indices included in this study: proportion of polymorphic fragments (PPOL), Shannon’s diversity index (SI), proportion of private fragments (PPRIV) and rarity index (RI). Estimated marginal means are represented by horizontal lines. The vertical bars are confidence intervals for the EMMs, and the red arrows are for the comparisons among them. Statistically significant differences (*P* < 0.05) are indicated by non-overlapping arrows.

Results of the linear and generalized mixed models (Table 3) indicated a significant effect of the type of marker in all diversity indices considered, but that these differences among markers fluctuated between restricted and widespread species as revealed by the significant ‘Markers-by-Distribution’ interaction term in all of them. Pairwise comparisons for each marker between restricted and widespread species of estimated marginal means (Fig. 4) revealed a complex pattern for most of the diversity indices. For instance, the proportion of polymorphic fragments was always significantly higher for M-MSAP than for AFLP or U-MSAP markers in both restricted (*Z* = 12.09, *P* < 0.0001; and *Z* = 8.060, *P* < 0.0001) and widespread species (*Z* = 11.94, *P* < 0.0001; and *Z* = 12.35, *P* < 0.0001). However, opposing trends were found between restricted and widespread species when U-MSAP and AFLP markers were compared. Specifically, U-MSAP markers had higher values of polymorphic fragments than AFLP markers (*Z* = 2.75; *P* = 0.0164) in restricted species, but the opposite pattern occurred in widespread species (*Z* = 2.62; *P* = 0.0241; Fig. 4). Similarly, proportion of private fragments (PPRIV) were consistently higher for AFLP or U-MSAP than for M-MSAP markers in restricted (*Z* =9.38, *P* < 0.0001; and *Z* = −8.06, *P* < 0.0001) and widespread species (*Z* = 8.79, *P* < 0.0001; and *Z* = 12.35, *P* < 0.0001). However, U-MSAPs had more private markers than AFLPs only in widespread species (*Z* = 2.62, *P* = 0.0241) but not in restricted species (Fig. 4). No significant difference in Shannon’s index (SI) was noted among the three different types of markers in restricted species, and also when each type of marker was compared between restricted and widespread species. Only in widespread species SI had slightly lower values for U-MSAP markers than for AFLPs (*t* = 2.73, *P* = 0.0173) and M-MSAP markers (*t* = 3.02, *P* = 0.0071; Fig. 4). Similar patterns of variation in rarity index (RI) were found among the three types of markers when comparing the two groups of species (Fig. 4). Actually, in both restricted and widespread species RI was always significantly higher in AFLPs than in U-MSAPs (*t* = 4.64, *P* < 0.0001 and *t* = 8.50, *P* < 0.0001) or in M-MSAPs (*t* = 2.49, *P* = 0.0343; and *t* = 5.80, *P* < 0.0001), and significantly lower in U-MSAPs than in M-MSAPs (*t* = 2.34, *P* = 0.0506 and *t* = 8.50, *P* < 0.0001). In summary, we were able to detect significant differences between restricted and widespread only in one group of epigenetic markers (U-MSAP), particularly for PPOL and PPRIV, but not in genetic markers or in epigenetic methylated markers (M-MSAP).

**Table 3.** Results of the linear and generalized mixed models examining the effect of the fixed predictors: type of Markers (AFLP, U-MSAP and M-MSAP), Distribution (restricted and widespread) and their interaction, on each of the four response variables, the diversity indices: proportion of polymorphic fragments, PPOL; Shannon’s diversity index, SI; proportion of private fragments, PPRIV; and rarity index, RI. Signif. codes: ***P < 0.001; **P < 0.01; *P < 0.05; ^•^*P* < 0.1.

Response variable	Predictors	χ ^2^	*P*
PPOL	Markers	325.41	<2.2e-16***
	Distribution	1.03	0.30958
	Markers * Distribution	15.79	0.00077***
SI	Markers	5.53	0.06284^•^
	Distribution	0.20	0.65303
	Markers * Distribution	4.99	0.08268^•^
PPRIV	Markers	207.66	<2.0e-16***
	Distribution	0.33	0.56618
	Markers * Distribution	7.61	0.02222*
RI	Markers	93.68	<2.2e-16***
	Distribution	0.36	0.54758
	Markers * Distribution	9.57	0.00834**

### Relationship between genetic and epigenetic diversity within populations

**Figure 5.**
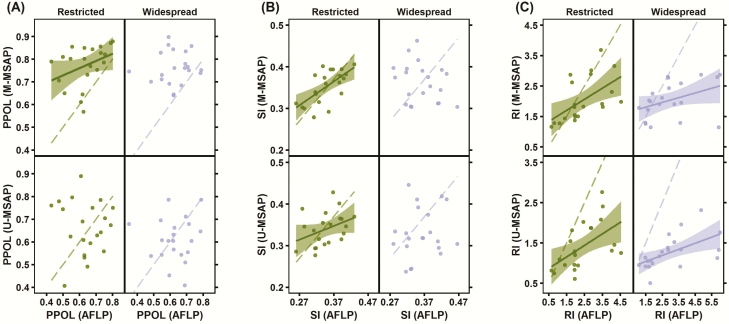
Pairwise relationships within populations between genetic (AFLP) and epigenetic (U-MSAP and M-MSAP) diversity indices for restricted and widespread species. Note that the relationship is illustrated only for three of the four indices that we have studied, in which at least one statistical significant correlation was found: (A) proportion of polymorphic fragments (PPOL); (B) Shannon’s diversity index (SI); and (C) rarity index (RI). Only statistically significant linear regressions (solid lines) and their 95 % confidence intervals (coloured area) were depicted. In all the figures the identity line (*x* = *y*) is also shown as a reference (dashed lines), and to emphasize that all points above these lines represent a greater epigenetic value compared to the same genetic value, whereas points below dashed lines indicate the reverse pattern.

In restricted species, populations that had more genetic diversity tended to have also more epigenetic diversity, as revealed by the statistically significant (or marginally significant) correlation between most of the diversity indices measured for AFLP markers, on one side, and on the other for the group of methylated (M-MSAP) markers (*r* = 0.374, 0.653 and 0.555; and *P* = 0.095, 0.0013 and 0.0089, for PPOL, SI and RI, respectively), or for the group of unmethylated (U-MSAP) markers (*r* = 0.372 and 0.540; P = 0.096 and 0.0113, for SI and RI, respectively; Fig. 5). However, in widespread species genetic and epigenetic diversity of study populations were largely independent of each other, as shown by statistically non-significant correlations between three of the four diversity indices measured for AFLP markers (PPOL, PPRIV and SI), and M-MSAP (*r* = 0.104, 0.118 and −0.267; and *P* = 0.65, 0.61 and 0.24); or U-MSAP type of markers (*r* = 0.044, 0.084 and 0.181; and *P* = 0.848, 0.716 and 0.431, respectively, for PPOL, PPRIV and SI; Fig. 5). Just RI in widespread species had significant correlation between genetic and epigenetic markers (*r* = 0.398 and *P* = 0.073 for the correlation between AFLP and M-MSAP markers; *r* = 0.563 and *P* = 0.007 for the correlation between AFLP and U-MSAP markers), concurring with the pattern found in restricted species (Fig. 5C).

## Discussion

Genetic diversity of natural populations is a fundamental trait in their evolutionary trajectory that summarizes past events and defines the potential for future adaptation to environmental changes (Jump *et al.* 2009; Allendorf 2017). Epigenetic variation, in turn, results from genetic and environmental factors, as well as from stochastic epimutations (Richards 2006; Johannes and Schmitz 2019), and it can also shape the evolutionary trajectories of populations (e.g. Kronholm and Collins 2016; Smithson *et al.* 2019). Epigenetic diversity can enhance plant population response under environmental stress (e.g. Latzel *et al.* 2013) but its magnitude and association with genetic diversity in natural populations is still poorly understood (Herrera *et al.* 2016; Moler *et al.* 2018). The concurrent analysis of the genetic and epigenetic diversity of 14 species presented here illustrates the wide range of genetic and epigenetic diversities that plant populations may harbour within a relatively small area of well-preserved montane habitats. In the next sections we will discuss the patterns obtained and how the absence of global consistent relationships between estimates of genetic and epigenetic diversity might be interpreted as the variable outcome of multiple determinants of genetic diversity (Leimu *et al.* 2006; Stuessy *et al.* 2014; Ellegren and Galtier 2016) and possibly other factors that were not accounted for in our study design.

### Genetic diversity

Endemic species account for the most original, phylogenetically distinctive component of local floras and a good portion of the rich biodiversity associated to hotspots (Médail and Quézel 1999; Melendo *et al.* 2003). Consequently, the magnitude of genetic diversity of endemic and threatened plant species has been investigated for decades with the dual interest of understanding their evolution and contributing to their conservation (Allendorf 2017). At a global geographic scale, pioneer analyses based on allozyme markers found lower percentages of polymorphic loci in endemic plants compared to widespread congeners (Karron 1987; Gitzendanner and Soltis 2000; Cole 2003). Likewise, López-Pujol *et al.* (2009) found a similar trend within a Mediterranean region, although without specifically controlling for relatedness due to scarcity of available congeneric comparisons. Later studies based on large numbers of anonymous DNA markers (e.g. AFLP, RAPD) found only partial support for this trend and confirmed the need to control for phylogenetic relatedness (Reisch and Bernhardt-Römermann 2014; Ellegren and Galtier 2016; and references therein). In the Mediterranean region, many narrow endemic plants of Western European mountains bear moderate to high levels of genetic diversity (Jiménez-Mejías *et al.* 2015), suggesting that divergence in genetic diversity between endemic and widespread species could not occur in this region (Forrest *et al.* 2017; and references therein). We are not aware, however, of any previous analysis addressing comparatively the genetic diversity in several congeneric pairs of endemic and widespread species at a local scale (i.e. simultaneously accounting for species relatedness and geographic location) in the Mediterranean or elsewhere.

In this study, the average polymorphism in AFLP markers per population ranged between 52.9 % (*D. laureola*, widespread) and 75.7 % (*E. cazorlanum*, restricted). All species studied here thus exceeded the population average value (41.6 % ± 21.5) reported by Reisch and Bernhardt-Römermann (2014) for 75 AFLP studies worldwide. These data suggested that our study region might be particularly rich not only in species numbers but also in the genetic diversity they preserve, which provides an extra value for future persistence (Jump *et al.* 2009). Previous studies in the Sierra de Cazorla region have likewise revealed consistently high levels of genetic diversity in plants and associated microfungi (Jordano and Godoy 2000; Medrano and Herrera 2008; Cánovas *et al.* 2015; Herrera *et al.* 2011, 2014), thus suggesting that this might be a distinctive feature of this biodiversity hotspot and highlighting the interest of conducting additional research in the framework of the ‘genetic hotspot’ concept (Souto *et al.* 2015).

Genetic distinctiveness of populations is also a relevant parameter from a conservation perspective (Jump *et al.* 2009; López-Pujol *et al.* 2009; Allendorf 2017). Except for one outlier population of *D. oleoides*, the PPRIV was <25 % in all populations and <10.7 % in half of them. We did not find significant differences between average PPRIV per population or RI of endemic and widespread plants at the local scale investigated here. Altogether our results supported the hypothesis that plant endemics from Western Mediterranean mountains do not necessarily harbour lower levels of genetic diversity (Forrest *et al.* 2017), although similar analyses in other geographic locations are required to verify the universality of this pattern. Furthermore, a wider geographic sampling should be conducted to conclude about population differentiation at the species level for conservation purposes. First, because genetic distinctiveness may appear only in a few populations that might be particularly valuable for the survival of endemic and widespread species (e.g. Hampe and Petit 2005; Forrest *et al.* 2017). And second, because in widespread species the few populations sampled in this study mostly occur at the rear-edge border of their distribution ranges, where genetic diversity could be particularly reduced or skewed and non-representative for the species (Hampe and Petit 2005; Eckert *et al.* 2008; Reisch and Bernhardt-Römermann 2014).

### Relationships between genetic and epigenetic diversity

We investigated epigenetic diversity associated to changes in the methylation status of hundreds of MSAP markers and compared it to genetic diversity estimates for roughly similar numbers of AFLP markers in 42 populations and seven pairs of congeneric plant species occurring sympatrically in the same geographic region. To the best of our knowledge this is the first study that has used a multispecies framework and a population-level approach to compare epigenetic variation and its relation with genetic variation of wild plants in nature (but see Liu *et al.* 2015 for a study with animals). In recent years some advances have been made in the emerging field of population epigenetics (see Kilvitis *et al.* 2014 for a review), which addresses questions about the prevalence and importance of epigenetic variation in the natural world (Richards 2008), but for plants most of these advances came from microevolutionary studies performed on single species and populations along ecological gradients, with unusual environmental exposures, or under invasive or range expansion scenarios (e.g. Herrera and Bazaga 2010; Lira-Medeiros *et al.* 2010; Richards *et al.* 2012; Schulz *et al.* 2014; Preite *et al.* 2015; Wilschut *et al.* 2016; Shi *et al.* 2019). Working in a multispecies framework we were searching for more general patterns so that our findings could provide valuable insight to fully understand the epigenetic phenomena under a broader evolutionary scale. In the group of our study species we have found that methylation polymorphism at cytosines that are frequently methylated (M-MSAP) ranged between 67.8 and 86.4 % (in restricted *V. cazorlensis* and *E. cazorlanum*, respectively), and was higher than polymorphism in those cytosines that are most commonly unmethylated (U-MSAP), which varied between 52.0 and 76.5 % (in restricted *T. rotundifolium* and *V. cazorlensis*, respectively). Epigenetic diversity was thus lower for U-MSAP than M-MSAP markers, and populations of endemic plants were more variable as regards the two epigenetic markers. Contrary to our expectations, however, populations of endemic and widespread species did not differ in their average epigenetic diversity. Although our sampling design simultaneously accounted for species relatedness and geographic location we cannot discard however that other factors important for plant population evolutionary dynamics such as population size or isolation level, which were not taken into account in our study, must also be operating and could have influenced our epigenetic (as well as genetic) diversity estimates. Recent models suggest, for instance, that greater rates of spontaneous epimutations can increase adaptation to local conditions and can help maintain polymorphisms in small and peripheral populations (Smithson *et al.* 2019). However, direct evidence of the inheritance of environmentally induced changes in DNA methylation in wild populations is still scarce. Some experimental reports indicate that stress-induced methylation changes can be inherited for one or two generations in the form of a ‘stress memory’ even in the absence of the stressor, but are quickly reverted to the non-stressed state thereafter (Wibowo *et al.* 2016; Lämke and Bäurle 2017). And, even under controlled experimental conditions, few studies have had the necessary experimental design to assess whether stress may also generate stable epimutations across multiple generations. In addition to this, recent studies have revealed that interspecific variation in plant epigenomic features is related also to other important intrinsic plant traits such as lifeform, as reflected by the fact that woody plants tend to have lower levels of global DNA methylation than perennial herbs (Alonso *et al.* 2019). Interestingly, three out of the four congeneric comparisons with strongest divergence in population epigenetic diversity involved pairs of herbaceous and woody congeners (*Anthyllis*, *Erodium*, *Viola*), although the sign of divergence was not consistent. Such intriguing results suggest that a deeper analysis with more species within genera but differing in other traits such as habitat requirements, lifeform, population size or isolation could be helpful to better understand the effect of species-specific plant traits in epigenetic diversity as previously suggested for genetic diversity (e.g. Gitzendanner and Soltis 2000).

Despite the heterogeneity of our study populations in the features mentioned above, we were still able to find significant relationships between genetic and epigenetic diversity, that were clearest for M-MSAP markers. Our results supported the expectation of higher epigenetic (M-MSAP) than genetic (AFLP) polymorphism in the whole sample, which was consistent with the idea that substantial epigenetic variation at shorter spatial distances might contribute significantly to population epigenetic diversity (see also Medrano *et al.* 2014; Herrera *et al.* 2016; and references therein). An excess of DNA methylation variation relative to genetic variation has been found in populations of the great majority of plant species examined to date and epigenetic variation has been linked to functional phenotypic variation (e.g. Gao *et al.* 2010; Herrera and Bazaga 2010; Lira-Medeiros *et al.* 2010; Richards *et al.* 2012; Medrano *et al.* 2014; Jueterbock *et al.* 2019; Shi *et al.* 2019; Wang *et al.* 2020), and also in most wild animals (for a recent review, see Hu and Barret 2017). In particular, Wang *et al.* (2020) found that the introduced populations of the clonal herb *Hydrocotyle vulgaris* exhibited extremely low genetic diversity but variation in specific leaf area and other leaf traits were all positively related to intra-population polymorphism in MSAP markers. Also, in clonal meadows of *Zostera marina* methylation variation promotes variation in fitness-related traits of ecological relevance, specifically photosynthetic performance and heat stress resilience, and contributes to the long-term survival of its genetically depauperate populations by increasing mitotically heritable and ecologically relevant phenotypic variation (Jueterbock *et al.* 2019). Such findings suggest that extensive epigenotypic variations may support phenotypic variation and have a key role in the evolutionary potential of wild populations, as predicted by theoretical models (e.g. Feinberg and Irizarry 2010; Furrow and Feldman 2014; Smithson *et al.* 2019). Moreover, for the group of endemic plants studied here the difference between epigenetic and genetic diversity was greater at populations with low genetic polymorphism, and the two variables were positively related, suggesting that at least in part epigenetic variation could be dependent on genetic variation. However, in widespread species, epigenetic polymorphism was consistently high regardless of the genetic polymorphism observed, and the two variables were not significantly related. Such contrasting patterns were partly consistent with our expectation that a higher epigenetic diversity could possibly alleviate the lower genetic diversity expected in populations of endemic plants. However, the relationship between the two variables remained positive and held at the population but not at the species level, suggesting that intraspecific variation was as relevant as interspecific variation in understanding the association between genetic and epigenetic diversities of wild plants (Herrera *et al.* 2016). A more complete understanding of the evolutionary relevance of epigenetic variation and the contribution of epigenetics to phenotypic differentiation and plant adaptation to environmental changes will require additional research on the extent to which epigenetically induced responses are also under genetic control and contribute to population and species persistence (Richards 2006).

## Conclusions

We found that all populations studied exhibited moderate to high levels of genetic polymorphism. However, contrary to our expectations, populations of restricted endemic and widespread species did not differ in average genetic or epigenetic diversity in our study region. The concurrent estimation of genetic and epigenetic diversity in replicated population samples of each species confirmed that levels of polymorphism at frequently methylated cytosines (M-MSAP markers) were always higher than polymorphism at genetic (AFLP) markers in both restricted endemic and widespread species. Importantly, correlation analyses showed that only in the group of species with restricted distribution did populations with higher epigenetic diversity tend to have also higher levels of genetic variation, although the difference between epigenetic and genetic diversity was greater at populations with low genetic polymorphism. This result is partly consistent with our expectation that a higher epigenetic diversity could possibly alleviate the lower genetic diversity expected in populations of endemic plants. Further work is needed to understand the effects that other intrinsic plant traits (e.g. lifeform) and population features (e.g. size, isolation) may have in comparative multispecies patterns of genetic and epigenetic co-variation in this Mediterranean hotspot region or elsewhere.

## Supporting Information

The following additional information is available in the online version of this article—

**Table S1.** Primer combinations used for amplified fragment length polymorphism (AFLP) and methylation-sensitive amplified polymorphism (MSAP) protocols.

**Table S2.** Replicated samples and scoring error rates in the amplified fragment length polymorphism (AFLP) and methylation-sensitive amplified polymorphism (MSAP) protocols.

**Table S3.** Genetic and epigenetic diversity estimates obtained in each population for the 14 study species.

**Table S4.** Summary of the genetic and epigenetic diversity estimates for the group of restricted endemic and widespread species.

plaa013_suppl_Supplementary_Files

## Sources of Funding

Financial support for this study was provided by grants CGL2013-43352-P and CGL2016-76605-P from the Spanish Ministry of Economy and Competitiveness.

## Contributions by the Authors

C.A., C.M.H. and M.M. conceived the idea and designed the study; C.A. and M.M. gathered plant material from natural populations; E.L. and P.B. isolated all DNAs and performed all AFLP and MSAP analyses; M.M. performed data analysis and prepared all figures and tables. C.A., C.M.H. and M.M. wrote the manuscript. All the authors read and approved the manuscript.

## Conflict of Interest

None declared.
