# Supplementary material for: Comparative genetic and epigenetic diversity in pairs of sympatric, closely related plants with contrasting distribution ranges in south-eastern Iberian mountains
Source: AoB Plants. 2020 Apr 8;12(3):plaa013. doi: 10.1093/aobpla/plaa013 (PMC7246305; doi:10.1093/aobpla/plaa013)
Supplement: plaa013_suppl_Supplementary_Files [file plaa013_suppl_supplementary_files.pdf]

SUPPORTING INFORMATION

Table S1. Selective primer combinations used for AFLP and MSAP analyses with the seven genera included this study.

	Primer combination	
Gender	AFLP	MSAP
Anthyllis	*Mse*I + CGT / *Pst*I + AA	*Mse*I + CAT / *Hpa*II–*Msp*I + TT
	*Mse*I + CCT / *Pst*I + AT	*Mse*I + CTC / *Hpa*II–*Msp*I + TA
	*Mse*I + CAT / *Pst*I + AC	*Mse*I + CAC / *Hpa*II–*Msp*I + TC
	*Mse*I + CAT / *Pst*I + AG	*Mse*I + CCT / *Hpa*II–*Msp*I + TC
Aquilegia	*Mse*I + CGT / *Pst*I + AA	*Mse*I + CAT / *Hpa*II–*Msp*I + TT
	*Mse*I + CAC / *Pst*I + AT	*Mse*I + CTC / *Hpa*II–*Msp*I + TA
	*Mse*I + CTT / *Pst*I + AC	*Mse*I + CAC / *Hpa*II–*Msp*I + TC
	*Mse*I + CAT / *Pst*I + AG	*Mse*I + CCT / *Hpa*II–*Msp*I + TC
Convolvulus	*Mse*I + CAC / *Pst*I + AA	*Mse*I + CGC / *Hpa*II–*Msp*I + TA
	*Mse*I + CTT / *Pst*I + AT	*Mse*I + CTC / *Hpa*II–*Msp*I + TA
	*Mse*I + CTT / *Pst*I + AC	*Mse*I + CGT / *Hpa*II–*Msp*I + TG
	*Mse*I + CAT / *Pst*I + AG	*Mse*I + CCT / *Hpa*II–*Msp*I + TC
Daphne	*Mse*I + CAC / *Pst*I + AA	*Mse*I + CGC / *Hpa*II–*Msp*I + TC
	*Mse*I + CAC / *Pst*I + AT	*Mse*I + CTC / *Hpa*II–*Msp*I + TA
	*Mse*I + CAT / *Pst*I + AC	*Mse*I + CGA / *Hpa*II–*Msp*I + TG
	*Mse*I + CTC / *Pst*I + AG	*Mse*I + CTC / *Hpa*II–*Msp*I + TC
Erodium	*Mse*I + CGT / *Pst*I + AA	*Mse*I + CAT / *Hpa*II–*Msp*I + TT
	*Mse*I + CAC / *Pst*I + AT	*Mse*I + CGC / *Hpa*II–*Msp*I + TA
	*Mse*I + CTT / *Pst*I + AC	*Mse*I + CCT / *Hpa*II–*Msp*I + TC
	*Mse*I + CTC / *Pst*I + AG	
Teucrium	*Mse*I + CGC / *Pst*I + AA	*Mse*I + CCT / *Hpa*II–*Msp*I + TT
	*Mse*I + CCT / *Pst*I + AT	*Mse*I + CGC / *Hpa*II–*Msp*I + TA
	*Mse*I + CTT / *Pst*I + AC	*Mse*I + CAC / *Hpa*II–*Msp*I + TC
	*Mse*I + CTT / *Pst*I + AG	
Viola	*Mse*I + CCC / *Pst*I + AA	*Mse*I + CAC / *Hpa*II–*Msp*I + TC
	*Mse*I + CCT / *Pst*I + AT	*Mse*I + CTC / *Hpa*II–*Msp*I + TA
	*Mse*I + CTT / *Pst*I + AC	*Mse*I + CGT / *Hpa*II–*Msp*I + TG
	*Mse*I + CAT / *Pst*I + AG	*Mse*I + CCT / *Hpa*II–*Msp*I + TC

Table S2. Percentage of replicated samples and scoring error rates in the AFLP and MSAP analyses for each of the 14 study species.

	AFLP		MSAP	
	replicated samples (%)	error rate (%)	replicated samples (%)	error rate (%)
Anthyllis ramburii	14.7	2.92	20.0	2.84
Anthyllis vulneraria	16.0	3.03	28.0	4.85
Aquilegia p. cazorlensis	14.7	1.85	26.7	2.79
Aquilegia v. vulgaris	16.0	1.88	26.7	3.32
Convolvulus boissieri	12.8	1.82	26.9	3.29
Convolvulus arvensis	14.7	2.12	24.0	3.40
Daphne oleoides	14.7	1.05	25.3	3.10
Daphne laureola	16.0	1.42	56.0	4.11
Erodium cazorlanum	8.6	3.48	13.3	2.93
Erodium cicutarium	12.3	2.02	17.3	3.46
Teucrium rotundifolium	14.7	2.51	20.0	2.61
Teucrium similatum	16.0	1.87	28.0	2.89
Viola cazorlensis	16.0	1.66	29.3	3.18
Viola odorata	17.6	2.09	20.3	3.11
mean	11.2	2.12	25.8	3.28
sd	2.2	0.66	9.9	0.58

Table S3. Genetic (AFLP) and epigenetic (U-MSAP and M-MSAP) diversity estimates obtained in each population (POP) for the 14 study species. Values of total number of fragments (NF); proportion of polymorphic fragments (PPOL); Shannon's diversity index (SI), proportion of private fragments (PPRIV); and Rarity Index (RI) are included.

		AFLP					U-MSAP					M-MSAP				
Species	POP	NF	PPOL	SI	PPRIV	RI	NF	PPOL	SI	PPRIV	RI	NF	PPOL	SI	PPRIV	RI
Anthyllis ramburii[R]	anra1	147	0.707	0.36	0.095	1.96	86	0.628	0.35	0.186	1.29	121	0.752	0.39	0.050	1.54
	anra2	147	0.667	0.39	0.075	1.98	86	0.593	0.36	0.128	1.20	121	0.769	0.38	0.041	1.49
	anra3	147	0.748	0.34	0.143	1.94	86	0.558	0.30	0.128	0.95	121	0.785	0.37	0.116	1.80
Anthyllis vulneraria[W]	anvu1	234	0.620	0.28	0.107	2.87	101	0.604	0.30	0.178	1.32	204	0.843	0.40	0.029	2.77
	anvu2	234	0.590	0.32	0.081	2.84	101	0.604	0.30	0.168	1.38	204	0.863	0.40	0.015	2.80
	anvu3	234	0.731	0.29	0.167	3.65	101	0.604	0.33	0.158	1.33	204	0.858	0.37	0.025	2.59
Aquilegia p. cazorlensis[R]	aqca1	163	0.546	0.40	0.067	1.72	106	0.623	0.33	0.208	1.54	191	0.801	0.37	0.042	2.87
	aqca2	163	0.509	0.39	0.080	1.95	106	0.406	0.32	0.047	0.83	191	0.649	0.34	0.005	1.88
	aqca3	163	0.730	0.34	0.233	2.85	106	0.642	0.35	0.274	1.87	191	0.827	0.40	0.068	2.89
Aquilegia v. vulgaris[W]	aqvu1	151	0.669	0.32	0.073	1.92	83	0.542	0.45	0.157	0.99	152	0.684	0.44	0.072	2.25
	aqvu2	151	0.623	0.35	0.172	2.49	83	0.566	0.41	0.229	1.28	152	0.645	0.46	0.105	2.11
	aqvu3	151	0.550	0.35	0.033	1.63	83	0.542	0.39	0.120	1.05	152	0.711	0.42	0.026	1.72
Convolvulus boissieri[R]	cboi1	184	0.728	0.35	0.174	2.88	148	0.784	0.38	0.088	1.87	194	0.856	0.36	0.082	3.01
	cboi2	184	0.500	0.39	0.082	1.81	148	0.743	0.40	0.054	1.81	194	0.809	0.36	0.041	2.62
	cboi3	184	0.620	0.37	0.130	2.45	148	0.689	0.43	0.061	2.02	194	0.644	0.39	0.015	1.82
Convolvulus arvensis[W]	carv1	223	0.484	0.33	0.058	2.34	121	0.537	0.32	0.050	1.17	157	0.701	0.33	0.076	1.90
	carv2	223	0.695	0.43	0.076	2.92	121	0.711	0.41	0.074	1.71	157	0.834	0.38	0.070	2.43
	carv3	223	0.789	0.42	0.161	3.66	121	0.785	0.42	0.132	1.95	157	0.752	0.39	0.045	1.95
Daphne oleoides[R]	dole1	86	0.756	0.26	0.407	1.97	54	0.704	0.29	0.056	0.61	95	0.821	0.31	0.021	1.37
	dole2	86	0.430	0.36	0.058	0.81	54	0.759	0.31	0.056	0.74	95	0.789	0.29	0.011	1.27
	dole3	86	0.477	0.33	0.012	0.66	54	0.778	0.32	0.093	0.81	95	0.705	0.32	0.000	1.16
Daphne laureola[W]	dlau1	114	0.693	0.36	0.184	1.78	59	0.508	0.32	0.068	0.50	145	0.779	0.39	0.028	1.99
	dlau2	114	0.544	0.32	0.149	1.70	59	0.695	0.33	0.169	0.91	145	0.828	0.39	0.028	2.02
	dlau3	114	0.351	0.42	0.061	1.08	59	0.678	0.28	0.169	0.95	145	0.745	0.34	0.014	1.78

Erodium cazorlanum[R]	ecazF	162	0.790	0.43	0.117	2.15	104	0.673	0.37	0.087	0.94	147	0.871	0.41	0.027	1.50
	ecazL	162	0.679	0.41	0.031	1.11	104	0.750	0.35	0.106	1.09	147	0.844	0.38	0.020	1.27
	ecazT	162	0.802	0.39	0.043	1.41	104	0.750	0.36	0.115	0.95	147	0.878	0.39	0.034	1.43
Erodium cicutarium[W]	ecicC	140	0.550	0.41	0.043	1.49	84	0.607	0.31	0.071	0.92	100	0.730	0.31	0.080	1.25
	ecicF	140	0.714	0.41	0.121	1.49	84	0.679	0.29	0.155	1.13	100	0.750	0.31	0.110	1.29
	ecicT	140	0.621	0.47	0.107	1.81	84	0.631	0.30	0.107	1.06	100	0.640	0.30	0.070	1.14
Teucrium rotundifolium[R]	trot1	319	0.630	0.32	0.135	4.18	100	0.540	0.29	0.230	1.45	186	0.849	0.38	0.081	3.15
	trot2	319	0.649	0.32	0.154	4.55	100	0.490	0.29	0.210	1.25	186	0.731	0.33	0.027	1.98
	trot3	319	0.630	0.32	0.107	4.03	100	0.530	0.28	0.210	1.30	186	0.801	0.34	0.032	2.31
Teucrium similatum[W]	tsim1	418	0.708	0.32	0.132	6.01	130	0.554	0.24	0.192	1.76	213	0.765	0.31	0.052	2.86
	tsim2	418	0.627	0.33	0.084	4.86	130	0.662	0.24	0.277	2.31	213	0.761	0.30	0.061	2.86
	tsim3	418	0.689	0.33	0.117	5.85	130	0.408	0.24	0.092	1.13	213	0.761	0.30	0.056	2.79
Viola cazorlensis[R]	vcaz1	261	0.556	0.31	0.103	3.42	181	0.796	0.34	0.011	2.08	185	0.854	0.28	0.227	3.68
	vcaz2	261	0.609	0.29	0.111	3.49	181	0.890	0.35	0.094	2.76	185	0.611	0.30	0.043	1.91
	vcaz3	261	0.625	0.28	0.123	3.52	181	0.608	0.39	0.039	2.40	185	0.568	0.30	0.059	1.81
Viola odorata[W]	vodo1	316	0.595	0.38	0.076	3.60	93	0.452	0.37	0.043	0.91	107	0.692	0.38	0.019	1.29
	vodo2	316	0.592	0.39	0.057	3.24	93	0.785	0.33	0.194	1.52	107	0.897	0.35	0.131	1.90
	vodo3	316	0.785	0.37	0.244	5.94	93	0.645	0.32	0.108	1.33	107	0.738	0.34	0.047	1.14

[R] species with restricted distribution; [W] species with widespread distribution

Table S4. Summary of the genetic (AFLP) and epigenetic (U-MSAP and M-MSAP) diversity estimates obtained across populations for the two groups of restricted-endemic and widespread species. Values of proportion of polymorphic fragments (PPOL); Shannon's diversity index (SI), proportion of private fragments (PPRIV); and Rarity Index (RI) are included.

		AFLP				U-MSAP				M-MSAP			
Distribution		PPOL	SI	PPRIV	RI	PPOL	SI	PPRIV	RI	PPOL	SI	PPRIV	RI
Restricted	mean	0.638	0.349	0.118	2.42	0.663	0.340	0.118	1.42	0.772	0.352	0.050	2.04
	se	0.023	0.010	0.018	0.24	0.026	0.009	0.016	0.13	0.020	0.009	0.011	0.16
	min	0.430	0.261	0.012	0.66	0.406	0.277	0.011	0.61	0.568	0.278	0.000	1.16
	max	0.802	0.430	0.407	4.55	0.890	0.428	0.274	2.76	0.878	0.405	0.227	3.68
Widespread	mean	0.629	0.361	0.110	3.01	0.609	0.329	0.139	1.27	0.761	0.363	0.055	2.04
	se	0.022	0.011	0.120	0.34	0.021	0.013	0.013	0.09	0.016	0.010	0.007	0.13
	min	0.351	0.280	0.033	1.08	0.408	0.237	0.043	0.50	0.640	0.303	0.014	1.14
	max	0.789	0.466	0.244	6.01	0.785	0.446	0.277	2.31	0.897	0.462	0.131	2.86
